# Repeat: a framework to assess empirical reproducibility in biomedical research

**DOI:** 10.1186/s12874-017-0377-6

**Published:** 2017-09-18

**Authors:** Leslie D. McIntosh, Anthony Juehne, Cynthia R. H. Vitale, Xiaoyan Liu, Rosalia Alcoser, J. Christian Lukas, Bradley Evanoff

**Affiliations:** 1 Department of Pathology and Immunology, Washington University in St. Louis School of Medicine, 660 S. Euclid, Box 8118, St. Louis, MO 63110 USA; 2 University Libraries, Washington University in St. Louis, 1 Brookings Drive, Campus Box 1061, St. Louis, MO 63130 USA; 3 Institute of Clinical and Translational Sciences, Washington University in St. Louis School of Medicine, 660 S. Euclid Ave, Box 8066, St. Louis, 63110 MO USA

**Keywords:** Reproducibility, Replication, Accessibility, Transparency, Electronic health records, Ehr, Secondary data re-use

## Abstract

**Background:**

The reproducibility of research is essential to rigorous science, yet significant concerns of the reliability and verifiability of biomedical research have been recently highlighted. Ongoing efforts across several domains of science and policy are working to clarify the fundamental characteristics of reproducibility and to enhance the transparency and accessibility of research.

**Methods:**

The aim of the proceeding work is to develop an assessment tool operationalizing key concepts of research transparency in the biomedical domain, specifically for secondary biomedical data research using electronic health record data. The tool (RepeAT) was developed through a multi-phase process that involved coding and extracting recommendations and practices for improving reproducibility from publications and reports across the biomedical and statistical sciences, field testing the instrument, and refining variables.

**Results:**

RepeAT includes 119 unique variables grouped into five categories (research design and aim, database and data collection methods, data mining and data cleaning, data analysis, data sharing and documentation). Preliminary results in manually processing 40 scientific manuscripts indicate components of the proposed framework with strong inter-rater reliability, as well as directions for further research and refinement of RepeAT.

**Conclusions:**

The use of RepeAT may allow the biomedical community to have a better understanding of the current practices of research transparency and accessibility among principal investigators. Common adoption of RepeAT may improve reporting of research practices and the availability of research outputs. Additionally, use of RepeAT will facilitate comparisons of research transparency and accessibility across domains and institutions.

**Electronic supplementary material:**

The online version of this article (doi:10.1186/s12874-017-0377-6) contains supplementary material, which is available to authorized users.

## Background

The reproducibility of research is of significant concern for researchers, policy makers, clinical practitioners and the public nationwide [1, 2]. Reproducibility, as defined by Stodden, Leisch, and Peng (2014) [3] is the calculation of quantitative scientific results by independent scientists using the original datasets and methods. This definition has been further distinguished into three types: computational reproducibility, empirical reproducibility [4], and replicability. Empirical reproducibility states that there is enough information available to re-run the experiment as it was originally conducted.

Recently, high-profile research integrity, data quality, or replication disputes have plagued many scientific disciplines including climate science, biomedical sciences, and psychology [5, 6]. These incidents have increased public and discipline community demands for research that is transparent and replicable. But conducting good science is challenging. The emergence of larger resources of data, the greater reliance on research computing and software, and the increasing complexity of methodologies combining multiple data resources and tools has characterized much of the current scientific landscape. The intersection of these advancements demonstrates the need for accessible and transparent science while simultaneously complicating the execution and traceability of reproducible research. Reproducibility in the biomedical research domain is no less challenging and important, given the clinical and health implications.

To support verifiable science, the practices and processes for true reproducibility must extend beyond the methods section of a journal article to include the full spectrum of the research lifecycle: analytic code, scientific workflows, computational infrastructure, other supporting documentation (e.g., specific time-stamped repository and database queries), research protocols, metadata, and more [7]. It is only with this well-curated information that research can be appropriately validated for transparency.

The goal of this project is to expand the focus of reproducibility to include more stages of the research data lifecycle as well as background aspects of the research environment. Specifically, we developed an empirical reproducibility framework to define elements needed to attempt to reproduce biomedical research. To limit the scope of this initial version so the project could be manageable yet scalable, we focused on: i) assessing the reproducibility of a study based on the publically available data and reported methodology and ii) limiting the variability of information presented in manuscripts by focusing on one biomedical research area - electronic health records (EHR). *Thus, we posit that through the developed framework using EHR-based research studies, we will have identified the elements needed to make a study empirically reproducible, as well as assess what gaps persist in existing publications and shared materials.*

## Methods

We used a multi-phase methods approach to determine the components needed for reproducing biomedical secondary data analysis studies based on EHR data. For this project we: i) Conducted a literature review to identify, code and summarize the required components for making research more reproducible; ii) Generated and refined a reproducibility framework; and, iii) Used a sample of manuscripts to test and refine the framework for face validity.

The team included: a director of clinical informatics (LMc), research data librarian (CHV), graduate students in clinical informatics and biostatistics (AJ, RA, XL), computer science intern (JCL), and the director of WU Institute for Clinical and Translational Sciences (BE). This study was approved by the Washington University in St. Louis Institutional Review Board.

### Identifying components for reproducible research

The first phase of this project involved defining components for research reproducibility (RR) through understanding the current landscape of biomedical research and exploring existing recommendations for making biomedical research more reproducible. The components for proposed best practices and recommendations for RR were identified and extracted using the following questions to guide our search:

- Hypothetically, if we conduct a methodological or meta-analytic review of the reproducibility of current practices within biomedical sciences, what information do we need to gather from the literature?
- How do the broad steps across the research life cycle gathered through current reproducibility research in other fields scale and manifest within the biomedical sciences?

**Fig. 1:**
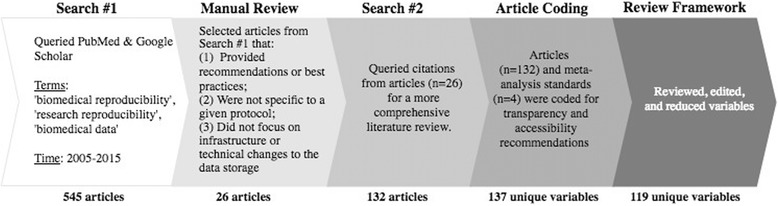
Workflow to identify elements needed to reproduce studies

As shown in Fig. 1, we searched PubMed and Google Scholar to identify English-language studies from 2005 to 2015 inclusive using the following terms: ‘biomedical reproducibility’, ‘research reproducibility’, and ‘biomedical data’ using the following syntax: “biomedical reproducibility”[All Fields] OR “research reproducibility” [All Fields] OR “biomedical data” [All Fields] AND (“2005/01/01”[PDat]: “2015/12/01”[PDat]) on December 2, 2015. Results returned 545 records - default sorted, and bibliographic information for these results was exported and then reviewed for exclusion criteria. Articles were excluded for multiple reasons including: (1) article did not provide recommendations or best practices; (2) were too specific to a given protocol; (3) focused on technical changes to the data store (*n* = 26). From this concentrated list, we expanded the relevant publications through searching cited references within these results to capture additional recommendations across biostatistics, data science, and health informatics [8–12]. A full list of literature collected during literature review search #2 used to define essential elements of reproducibility throughout the development of the RepeAT framework can be found in Additional file 1. We then reviewed protocols for conducting meta-analyses and systematic reviews from the Cochrane Collaboration [13], Joanna Briggs Institute [14], Institute of Medicine of the National Academies [15], and the Preferred Reporting Items for Systematic Review and Meta-Analysis protocols group [16] (*n* = 132). We compared these meta-analysis standards to determine additional elements for data extraction. Procedures for ensuring accuracy, consistency, and reliability throughout the development and implementation of study data collection, review, and analysis methods were coded from materials and were then adjudicated by the study team for appropriate implementation within study protocols.

### Empirical reproducibility framework development for biomedical research

Leveraging the literature review framing the broad components for biomedical RR, we then identified specific elements for the RR framework. These are elements across the research lifecycle both previously mentioned in the RR literature and unique to biomedical research.

The items were developed in consultation with existing documentation and publications on data management, data sharing, and implementing reproducible research. The existing standards and best practices within the field used in defining variables of interest included: *The NISO Primer: Research Data Management* [17]; *Data Management for Researchers: Organize, maintain and share your data for research success* [18]; the New England Collaborative Data Management Curriculum [19]; and, recommendations developed through the Research Data Alliance, in particular the Citation of Evolving Data recommendations [20]. In anticipation of future analyses, the study team then tagged the items in two ways: i) transparency of the research workflow; and, ii) accessibility of shared information (Table 1).

**Table 1:** Categories for RR variables

Axes of Research Reproducibility	Example	Categories
Transparency is the robust write up or description of research, such that it is clear and explicit.	All data collection processes are described clearly within publication methods and metadata.	data collection, data cleaning/preparation, data integration, data analysis, data sharing, code (cleaning, integration, analysis), data, software, documentation
Accessibility is a multi-faceted term encompassing both sharing and discoverability. Shared information such as a research dataset or analysis code must be discoverable, in a form that people can use, and available. Discoverability is defined as being in a location that enables the finding of the data and supplemental materials.	A query script used in data collection procedures is shared in a freely accessible and easily discoverable database.	

### Testing the face validity of framework items

The project team refined the instrument using three approaches to evaluate the appropriateness of the items: i) iteratively reviewing items to clarify item meaning; ii) completing the items using published EHR studies to field test the instrument; and iii) assessing the inter-rater reliability.

#### Internal review of framework items

The Repeatability Assessment Tool (RepeAT) Framework underwent an internal review wherein three authors (LMc, CHV, AJ) independently reviewed the phrasing and structure of the questions and variables. These internal review phases aimed to evaluate the precision and specificity of question phrasing as well as avoid single items with multiple meanings (e.g., double-barreled questions). The overarching goal of the internal review process was to limit potential for information bias during data collection and refine a workflow for data entry. An additional goal was to enhance the likelihood of generalizability and ease of adoption wherein researchers unfamiliar with the concept of reproducibility or having cursory knowledge of data management could comprehend pertinent measures within the RepeAT framework.

A second round of internal testing was conducted wherein a sample of published research articles were run through the data collection framework (RepeAT) to evaluate variable pertinence and completeness. Edits were made to certain variables to document more granular study information that was not in keeping with reproducibility best practices, yet relevant for data collection and analyses. For example, the hypothesis variable could be labeled as “unclear” if not adequately stated instead of only “present” or “absent”, and for free text entry of ambiguous database titles such as “hospital records” or a similar account of an electronic medical record system. Such data entry provides study insight in lieu of a clear citation or description of study data sources. Additional items not in reproducibility best practices yet potentially relevant for future analyses were added to include study bibliographic information (e.g., journal name, author’s institution).

#### Identification of published studies for testing

Peer-reviewed, scholarly publications using EHR data for secondary analysis, published between 2011 and 2016, and searchable through PubMed Central were considered for testing RepeAT.

PubMed Central [21] was searched to identify English-language studies that had ‘electronic health records’ as a keyword from the period 2011–2016 using the following syntax: “electronic medical records”[All Fields] OR “electronic health records”[All Fields] AND (“2011/07/11”[PDat]: “2016/02/02”[PDat]) on February 2, 2016. Results returned 13,880 records - default sorted, and bibliographic information for these results was exported. Taking a sample of the results (*n* = 300), the team screened the titles and abstracts based on the following inclusion criteria in order to identify applicable studies:

1. Secondary analysis of EHR data (i.e., not EHR for recruiting a cohort nor meta-analysis of treatment literature using secondary EHR data)
2. Published in English
3. Published between 2011 and 2016
4. Human research
5. Use of digital extraction from health record (via data broker or query interface)

Articles were excluded for multiple reasons such as the research was about the EHR but did not use data from the EHR. Excluded articles and a reason for exclusion are documented in supplementary materials [22]. If the title and the abstract were not clear enough to determine whether or not to reject, the full-text of the article was retrieved and evaluated. Reviewers assessing the study eligibility were not blinded to the names of the authors, journals, and other publication details as these were critical elements to include in the tool. Duplicate studies were not included, and the first 40 articles in the sampled dataset fitting the inclusion criteria were selected for field-testing.

#### Inter-rater reliability

To further assess the face validity, three raters (XL, RA, JCL) completed RepeAT using 40 selected studies. Because of limitations in the number of raters available, as well as a small number of coded articles, we used this assessment as a guide in refining the items rather than a strict statistical test that would remove an item.

##### Raters

The raters had an educational background in biostatistics or computer science as well as multiple years experience working with clinical research data. Author (AJ) trained all raters on the RepeAT framework and provided a demonstration of data entry using a sample publication to clarify variable elements. Raters were free to raise questions or concerns if they could not comfortably understand a publication’s research domain or design, or to clarify how ambiguously reported methods ought to be entered within the tool. Raters were not blinded to the role of authors in the development of the tool, the names of other participating raters, or to the fact that their entered data would be used for inter-rater reliability. Raters were instructed to not compare or discuss individual publications or variables with one another in an attempt to minimize bias.

##### Data and analyses

A file of all records entered in RepeAT was exported from REDCap [23] in a comma separated (csv) format (Additional file 2: Inter-rater Reliability RepeAT Data). Records were then excluded if they were not reviewed by more than two raters. Analyses were conducted using R (Additional file 3: Inter-rater Reliability Analysis Code) with R packages (psych, dplyr, tydr) [24–28].

The percent of observed agreement and inter-rater reliability between both raters was assessed using Cohen’s kappa [29]. We are using the standard Landis and Koch magnitude guidelines to interpret the percent agreement of Cohen kappa statistics (> 0.8 – almost perfect; 0.8–0.6 – moderate; 0.4–0.2 – fair, 0.2–0.0 – slight; < 0 no agreement [30] . To satisfy test criteria, all variables included in the analysis were nominal or binary. In addition, multi-select nominal variables were excluded from analysis. To see the full list of variables examined, please refer to Additional file 4: Appendix. Tests of Cohen’s kappa were conducted against the null hypothesis that the proportion of agreement between raters was not significantly larger than the proportion of agreement expected by chance. To satisfy test assumptions, variables having a variance of zero for both raters were excluded from the Cohen’s kappa analysis.

## Results

### Components for reproducible research

From the articles collected from the previous step, the study team recorded the practices and recommendations for reproducing research based on:

i) Steps within the research data lifecycle – data collection, data cleaning/preparation, data integration, data analysis, and data sharing
ii) Software code – scripts and queries used for data cleaning, integration, analysis
iii) Versions of data produced throughout the research process
iv) Versions of software and hardware used throughout the original research study
v) Documentation and metadata of analysis materials and relevant shared deliverables of the research output

### Face validity of framework variables

To test and refine the RepeAT framework, we assessed the face validity of each item that had a single categorical response (*n* = 75). Bibliographic data including author name, digital object identifier (DOI) or PubMed Central reference number (PMCID), publication title, and reproducibility characteristics (Table 2) were extracted from the articles and coded through a manual review of the publication and linked supplemental materials (Additional file 2: Inter-rater Reliability RepeAT Data). The study team members coded responses only from information contained in the published articles or referenced as an outside supplemental document; we did not contact the authors or investigators directly for missing information.

**Table 2:** RepeAT framework variables where inter-rater reliability could be calculated using Cohen’s kappa

RepeAT Framework Variable	Cohen’s Kappa	Kappa Bounds	var Rater 1	var Rater 2	Percent Agreement
Publication state database(s) source(s) of data?	0.320	(0.580–0.060)	0.095	0.250	70.6
Does the publication clearly state process(es) for validating data minded via nlp and/or queried from a database?	0.440	(0.860–0.019)	0.182	0.069	85.7
Does the author state any clear process documented for accounting for missing data?	0.520	(0.890–0.140)	0.115	0.261	83.3
Does the research involve natural language processing or text mining?	0.870	(1.100–0.630)	0.134	0.107	97.1
Does the author indicate the software used to develop the analysis code?	0.880	(1.000–0.710)	0.236	0.243	94.1

Where possible, Cohen’s kappa was used to assess inter-rater reliability. Five items meet criteria for statistically significant calculation of Cohen’s kappa (Table 2). At least one rater had a variance greater than zero and a large enough number of observations were entered by each rater to calculate the amount of agreement that would occur by chance.

The percent agreement between raters is calculated (Additional file 4: Appendix) for variables wherein Cohen kappa could not be calculated (*n* = 36) due to observed variance or numbers of observations. In most cases, this lack of variance between raters is due to a majority of manuscripts failing to meet criteria for the variable of interest. For example, for the question “is the finalized dataset shared?” there is 100% agreement between raters yet both raters have zero variance in their answers. We interpret this as both raters equally understanding the meaning of the question and having equal ability to assess the lack of pertinent information within the article or materials linked to the article to meet criteria for sharing data.

Variables with high rates of missing data (*n* = 36) prevented the option to calculate percent agreement or Cohen’s kappa (Additional file 4: Appendix). Missing data is most likely due to a majority of manuscripts failing to meet skip logic criteria built into the REDCap forms for preceding or linked questions. For example, as 100% of manuscripts were rated as failing to share a query script, the question “Does the shared query script for database 1 contain comments and/or notations for ease of reproducibility” was not displayed and thus barred from data entry. This high rate of missing data may also be due to ambiguous reporting practices across the selected manuscripts. Further research is required to assess how question phrasing or variable elements could be altered to provide options to note instances where it is unclear if a manuscript meets or fails criteria.

### Reproducibility framework for biomedical research

Based on the concepts in Table 3 and the face validity assessments, we selected, operationalized, and iteratively refined the variables identified from the literature review into specific process questions. Electronic case report forms (CRF) were then constructed within the REDCap application to have a structured database for data collection, storage, and export throughout the study period. As summarized in Table 4, the CRFs focused on five areas of reproducibility: Publication Overview and Bibliographic Information; Database and Data Collection; Methods: Data Mining and Cleaning; Methods: Data Analysis; Data Sharing and Documentation. Items have been flagged as pertaining to transparency or accessibility; however, this is only visible upon data export and not data entry. The final categories and a sample number of items included in the RepeAT framework are reported in Table 4 with the complete list in the Additional file 4: Appendix and online [22].

**Table 3:** RepeAT framework categories and concepts

Reproducibility Category	Major Concepts
Research Design and Aim	Recording administrative and study information
Database and Data Collection Methods	Clarifying study data source(s) and methods of collection
Data Mining and Data Cleaning	Describing process for cleaning, merging, and validating data
Data Analysis	Clarifying methods and materials for data analysis
Data Sharing and Documentation	Making relevant research data and documentation shared, accessible, and intelligible

**Table 4:** Abbreviated RepeAT framework with example variables^a^

Publication Overview and Bibliographic Information (21 items)	
Article Title	Text
DOI	Text
Is the research hypothesis-driven or hypothesis-generating?	Hypothesis Driven Hypothesis Generating Unclear
Database and Data Collection (63 items)	
Publication states database(s) source(s) of data?	Yes/No
^b^Publication states database(s) source(s) of data in the following location:	Not Stated Supplementary materials Body of Text
Query methodology	Manual extraction Digital extraction through query interface Digital extraction through honest broker Not Applicable/Not Stated
^b^Does the shared query script for database contain comments and/or notations for ease of reproducibility?	Yes/No
Methods: Data Mining and Cleaning (19 items)	
Does the research involve natural language processing or text mining?	Yes/No
^b^Please list all software applications used for text mining: * Please enter all that apply separated by a semi-colon*	Text
^b^Is the text mining software application proprietary or open? * If multiple applications were used, please select all options that apply.*	1. Proprietary 2. Mixed 3. Open
Methods: Data Analysis (15 items)	
Does the author state analysis methodology and process?	Yes/No
Does the author indicate the software used to develop the analysis code?	Yes/No
^b^Is the analysis software proprietary or open?	Proprietary Open
Data Sharing and Data Documentation (36 items)	
Is the finalized dataset shared?	Yes No
^b^Where is the finalized dataset shared?	Affiliated Research Center Website Author’s Institution or Department Website Data Registry Journal or Publication’s Website GitHub Other
Is there a clear process for requesting the data?	Yes No

^a^The full Framework can be found in the Additional file 4: Appendix as well as online within this project's Github repository (https://github.com/CBMIWU/Research_Reproducibility/tree/master/DataDictionary) and our project's Open Science Framework project management tool (Additional file 3: https://osf.io/ppnwa/)

^b^Indicates items that are shown only if a specific response to another item has been selected using skip-logic

## Discussion

### General

Having a reproducible study has many benefits including facilitating one’s own research through time (e.g., when staff leave), easing research dissemination, and allowing researchers to *not* reproduce or repeat a study if they do not wish, thus reducing inefficient use of research time, resources, or funding. Moving towards creating a list of the essential materials and process insights necessary for project reproducibility can aid in communicating what knowledge and documentation needs to be shared across collaborating project members with distinct roles across the data lifecycle.

Each of these distinct roles – such as informaticians, data analysts, clinicians, statisticians, database administrators, data and bio-curators – have unique sets of skills, processes, and difficulties throughout the transformation of data into study findings and accessibility. Improved sharing and documentation by parties involved across the research data lifecycle will likely result in more transparent study methods within a publication as well as a more robust and comprehensive package of materials to be shared alongside. The work described in this project is complementary to ongoing initiatives and expands the current focus of data management documentation towards encompassing scientific workflows and process management [36].

Though much has been written about the irreproducibility of research as just noted, little effort has been placed on developing assessment tools to determine the transparency of existing publications and the research it represents. The developed framework – RepeAT – integrates many agreed upon standards and best practices supported by leading scientific agencies, allowing one to measure how current biomedical science is meeting or lagging behind these recommendations. The framework also accounts for multiple stages of data management across the research data life cycle, a longitudinal perspective crucial to reproducibility though sometimes lacking in finalized published content.

Some observations of this work should be highlighted: 1) There are many variables in the RepeAT framework. As this is the first attempt to document items needed for an empirical reproducibility study, there may be too many items defined and/or it may be indicative that reproducibility is complex. We cannot tell without further investigation; 2) Many variables could not be analyzed for IRR because there are currently none reported in the literature; 3) Some refinement of the framework should occur. As noted, some variables are interesting for future analyses but not necessary for empirical reproducibility (e.g., author institution) and there are a few items with confusing wording; and, 4) Even though a sampled article does not have all the elements listed in a publication to make it reproducible does not mean the study cannot be reproduced. For example, a study may still be reproducible if the data, code, and protocols are widely shared, but are not significantly detailed in the publication.

Specific to the face validity analyses, it appears both raters were able to equally understand the meaning and intention of variables resulting in strong Kappa results (*n* = 2) or rater agreement >70% (*n* = 30). Such cases of high kappa may also point to manuscripts clearly reporting information pertinent to each variable, thus, allowing raters to ascertain reliably the presence or lack of information within the manuscript to satisfy each variable’s criteria. In such instances, both the wording of the question and the reporting practices of the manuscript are of adequately high clarity.

Variables resulting in moderate to weak kappa values (*n* = 3) or agreement between raters <70% (*n* = 11) demonstrate the need for further testing of the framework’s reliability through both expansion of the manuscript sample size and evaluation of variable wording. In essence, it is unclear if the wording of the framework variables is ambiguous, leading to conflicting interpretation on the part of the raters, or if the selected manuscripts report information pertinent to framework variables in an unclear manner. We are assessing this in our future research so we can either reduce or clarify variables as needed.

### Limitations

This study has multiple limitations. To make the study manageable, we limited the research to only those manuscripts that used EHR data for secondary data analysis, which represents only a small component of biomedical research.

The character of publications and associated data collected for inclusion in the sample of materials to be assessed using the framework is highly dependent upon the protocols of journal publishers, which may affect the way research is represented (e.g., journal word count limits, availability of supplemental file deposits). Despite these imposed limitations, we feel investigators still have a responsibility to document and support their research claims through other mechanisms, such as hosting data and code in open data repositories or providing open access to research methods. We also did not attempt to contact authors of reviewed papers to determine if missing elements could be obtained through direct contact. Hence, we cannot tell why a study may not be reproducible only if the elements are available to attempt to reproduce it.

Moreover, as the research team was small, this study and the elements defined were limited; more perspectives could be gained through having more team members, particularly at other institutions. We hope this limitation will be addressed through this publication as well as making RepeAT publically available for comments.

### Next steps

Field-testing of the RepeAT framework will continue to include a more robust sample of publications. A round of validation testing will also be conducted using the full sample of publications and a drafted ‘gold-standard’ publication that fulfills all reproducibility criteria within RepeAT. We are also developing a software tool to automate the assessment of these items.

A working version of the framework has been shared openly [22] specifically for the biomedical and data science communities to allow for discourse, criticism, and suggestion of the included variables forming the proposed characteristics and standards of reproducibility.

Furthermore, this study and framework does not suggest all research data and supporting documentation must be made openly and widely available for it to be replicable. We recognize in some situations, the data or code may be very sensitive or proprietary. Therefore, we posit that reproducibility should hinge more on the fact that robust documentation should exist rather than all data should be openly available. Hence, if given the proper access to the dataset, robust research protocols, and well-documented workflows, the research should be replicable. Limiting a dataset’s availability because of privacy issues (e.g., personally identifiable information, trade secrets) does not negate the potential for a particular study’s reproducibility. However, more work in this area needs to be completed.

## Conclusion

Though much work is leading the way in documenting reproducibility practices [31–34] successes, and failures, the essential characteristics defining reproducibility remain unclear. Explanation of the rational and method driving the RepeAT framework development and analysis of publications ought to provide a more inclusive dialogue concerning the current weakness in reproducibility, methods of operationalizing reproducibility concepts, and potential benchmarks of reproducibility that ought to be reached.

While, it is not a framework designed to assess the quality or rigor of all biomedical data or research; it does move the field forward to define the level of transparency and accessibility of associated research data, materials, and adopted resources. Thus we posit, through using this framework researchers (e.g., clinical scientists, informaticians, and informationists) can identify areas needed for an EHR study to be reproducible and develop practices or tools that may enhance the reproducibility of clinical research.

## Additional files

Additional file 1: Literature Review Search #2, Description: list of literature collected during literature review search #2 used to define essential elements of reproducibility throughout the development of the RepeAT framework. (RTF 117 kb)

Additional file 2: Inter-rater Reliability RepeAT Data; Description: data entered into the RepeAT framework by two independent raters and subsequently used for inter-rater reliability testing. (CSV 102 kb)

Additional file 3: Inter-rater Reliability Analysis Code; Description: programming script in R demonstrating data formatting, cleaning, inter-rater reliability analysis processes, and results. (R 130 kb)

Additional file 4: Appendix: RepeAT Framework, list of entirety of RepeAT framework variables with corresponding kappa values, percent rater agreement, and relation to transparency or accessibility. (DOCX 37 kb)

## Abbreviations

EHR: Electronic Health Records
RepeAT: The Repeatability Assessment Tool

## References

[1] Landis SC, Amara SG, Asadullah K, Austin CP, Blumenstein R, Bradley EW (2012). A call for transparent reporting to optimize the predictive value of preclinical research. Nature.

[2] Freedman LP, Inglese J. The Increasing Urgency for Standards in Basic Biologic Research. Cancer Res. 2014 [cited 2016 Nov 2]; Available from: http://cancerres.aacrjournals.org/content/early/2014/07/17/0008-5472.CAN-14-0925 10.1158/0008-5472.CAN-14-0925 PMC4975040 25035389

[3] Stodden V, Leisch F, Peng RD. Preface. Implement. Reprod. Res. [Internet]. Chapman and Hall/CRC; 2014. Available from: https://www.crcpress.com/Implementing-Reproducible-Research/Stodden-Leisch-Peng/p/book/9781466561595

[4] Stodden V. What Scientific Idea is Ready For Retirement? | Edge.org [Internet]. [cited 2016 Nov 2]. Available from: https://www.edge.org/annual-question/what-scientific-idea-is-ready-for-retirement

[5] Coombes KR, Wang J, Baggerly KA. Microarrays: retracing steps [Internet]. 2007. Available from: http://www.nature.com/nm/journal/v13/n11/full/nm1107-1276b.html 10.1038/nm1107-1276b 17987014

[6] Center for Open Science [Internet]. SHARE. 2015 [cited 2016 Nov 2]. Available from: http://www.share-research.org/about/our-team/center-for-open-science/.

[7] Khoury MJ, Lam TK, Ioannidis JPA, Hartge P, Spitz MR, Buring JE (2013). Transforming epidemiology for 21st century medicine and public health. Cancer Epidemiol Prev Biomark.

[8] Ioannidis JPA, Khoury MJ (2011). Improving validation practices in “Omics” research. Science.

[9] Ryan MJ (2011). Replication in field biology: the case of the frog-eating bat. Science.

[10] Tomasello M, Call J (2011). Methodological challenges in the study of primate cognition. Science.

[11] Ioannidis JPA (2014). How to make more published research true. PLoS Med.

[12] Ioannidis JPA, Khoury MJ (2014). Assessing value in biomedical research: the PQRST of appraisal and reward. JAMA.

[13] Cochrane Handbook for Systematic Reviews of Interventions [Internet]. [cited 2015 Dec 23]. Available from: http://handbook.cochrane.org/

[14] Peters M, Godfrey C, McInerney P, Soares C, Hanan K, Parker D. The Joanna Briggs Institute Reviewers’ Manual 2015: Methodology for JBI Scoping Reviews. 2015 [cited 2016 Nov 5]; Available from: http://espace.library.uq.edu.au/view/UQ:371443

[15] Standards for Systematic Reviews: Health and Medicine Division [Internet]. [cited 2016 Nov 5]. Available from: http://www.nationalacademies.org/hmd/Reports/2011/Finding-What-Works-in-Health-Care-Standards-for-Systematic-Reviews/Standards.aspx

[16] Moher D, Shamseer L, Clarke M, Ghersi D, Liberati A, Petticrew M (2015). Preferred reporting items for systematic review and meta-analysis protocols (PRISMA-P) 2015 statement. Syst Rev.

[17] Strasser C. Research Data Management [Internet]. National Information Standards Organization; 2015. Available from: http://www.niso.org/apps/group_public/download.php/15375/PrimerRDM-2015-0727.pdf. Accessed 12 July 2017.

[18] Briney K. Data Management for Researchers: Organize, maintain and share your data for research success. Exeter: Pelagic Publishing Ltd; 2015.

[19] New England Collaborative Data Management Curriculum | Lamar Soutter Library - University of Massachusetts Medical School [Internet]. [cited 2016 Nov 5]. Available from: http://library.umassmed.edu/necdmc/index

[20] Rauber A, Asmi A, van Uytvanck D, Pröll S. Data citation of evolving data:Recommendations of the Working Group on Data Citation (WGDC). Result of the RDA Data Citation WG, 2015. p. 2015.

[21] pubmeddev. PubMed - NCBI [Internet]. [cited 2016 Nov 2]. Available from: https://www.ncbi.nlm.nih.gov/pubmed

[22] CBMIWU/Research_Reproducibility [Internet]. GitHub. [cited 2016 Nov 2]. Available from: https://github.com/CBMIWU/Research_Reproducibility

[23] REDCap [Internet]. [cited 2016 Nov 2]. Available from: https://redcap.wustl.edu/redcap/srvrs/prod_v3_1_0_001/redcap/

[24] R: A Language and Environment for Statistical Computing [Internet]. Vienna, Austria: R Core Team; 2016. Available from: https://www.R-project.org/.

[25] Revelle W (2016). Psych: procedures for psychological, psychometric, and personality research [internet].

[26] Wickham H, Francois R. dplyr: a grammar of data manipulation. R package version 0.2. 2016. Available from: https://CRAN.R-project.org/package=dplyr.

[27] Wickham H. Easily Tidy Data with 'spread ()' and “gather ()” Functions. 0. CRAN R Package. http://cran/R-projectorg/package=tidyr. version. 2016;41.

[28] Warnes GR, Bolker B, Lumley T, Johnson RC. gmodels: Various R programming tools for model fitting. Includes R source code and/or documentation contributed by Bolker B, Lumley T, Johnson RC. R package version. 2006;2(0). Available from: https://CRAN.R-project.org/package=gmodels.

[29] Cohen J (1960). A coefficient of agreement for nominal scales. Educ Psychol Meas.

[30] Landis JR, Koch GG (1977). The measurement of observer agreement for categorical data. Biometrics.

[31] Rauber A, Miksa T, Mayer R, Proell S. Repeatability and Re-usability in Scientific Processes: Process Context, Data Identification and Verification. InDAMDID/RCDL 2015 Oct 13 (pp. 246-256). [cited 2016 Nov 2]; Available from: http://ceur-ws.org/Vol-1536/paper33.pdf

[32] Hettne KM, Wolstencroft K, Belhajjame K, Goble CA, Mina E, Dharuri H, et al. Best Practices for Workflow Design: How to Prevent Workflow Decay. SWAT4LS [Internet]. 2012 [cited 2016 Nov 2]. Available from: https://www.researchgate.net/profile/Kristina_Hettne/publication/258568127_Best_Practices_for_Workflow_Design_How_to_Prevent_Workflow_Decay/links/02e7e528d0ce01a135000000.pdf.

[33] wf4ever [Internet]. [cited 2016 Nov 2]. Available from: http://wf4ever.github.io/ro/.

[34] FAIRshairing [Internet]. [cited 2016 Nov 2]. Available from: https://fairsharing.org/standards/.

[35] SciDataCon [Internet]. [cited 2017 May 6]. Available from: http://www.scidatacon.org/2016/sessions/65/poster/34/.

