# Supplementary material for: Repeat: a framework to assess empirical reproducibility in biomedical research
Source: BMC Med Res Methodol. 2017 Sep 18;17:143. doi: 10.1186/s12874-017-0377-6 (PMC5604503; doi:10.1186/s12874-017-0377-6)
Supplement: Supplementary file 4 — Appendix: RepeAT Framework, list of entirety of RepeAT framework variables with corresponding kappa values, percent rater agreement, and relation to transparency or accessibility. (DOCX 37 kb) [file 12874_2017_377_MOESM4_ESM.docx]

**Appendix: RepeAT Framework
Full list of all RepeAT Framework variables, relation to transparency or accessibility, and associated percent inter-rater agreement where available data provided adequate significance for calculation**

| **Publication Overview and Bibliographic Information (21 items)** | | |  |
| --- | --- | --- | --- |
|  | Article DOI | Bibliographic |  |
|  | Article Title | Bibliographic |  |
|  | Corresponding Author Email | Bibliographic |  |
|  | Corresponding Author First Name | Bibliographic |  |
|  | Corresponding Author Last Name | Bibliographic |  |
|  | Publication states null and alternative hypothesis? | Transparency | 25.64% |
|  | Clinical Domain Category | Bibliographic |  |
|  | Project funders | Transparency |  |
|  | Grant identifiers | Transparency |  |
|  | Is the research hypothesis-driven or hypothesis-generating? | Transparency | 35.99% |
|  | Institution of Corresponding Author | Bibliographic |  |
|  | Journal of Publication | Bibliographic |  |
|  | Is the publication presenting a meta-analysis? | Transparency | 100% |
|  | Is the publication presenting primary research wherein new data is collected and/or secondary data is reused towards a new purpose? | Transparency | 61.11% |
|  | Publication Date | Bibliographic |  |
|  | Is the publication submitted as part of a special issue or editorial? If yes, please list the issue title. | Bibliographic |  |
|  | Record ID | Bibliographic |  |
|  | Is the publication presenting an attempt at reproducing research? | Transparency | 94.87% |
|  | Author's Affiliated Research Center | Bibliographic |  |
|  | Reviewer name | - |  |
|  |  |  |  |
| **Database and Data Collection (63 items)** | | |  |
|  | Publication states database(s) source(s) of data? | Transparency | 0.320 |
|  | Publication states database(s) source(s) of data in the following location: | Accessibility |  |
|  | Does the publication include database citation(s)? | Accessibility | 90.91% |
|  | Does the researcher share a query script for databases used throughout the data collection workflow? | Transparency & Accessibility | 100% |
|  | Do the authors state the methods used for collecting data? | Transparency & Accessibility | 83.33% |
|  | States query methodology in the following location: | Transparency |  |
|  | In the boxes below, please enter the full name or citation for each applicable database listed within the publication. If less than four databases are cited, please complete sections for all applicable databases and leave the subsequent fields blank. | Accessibility |  |
|  | Database Name 1 | Accessibility |  |
|  | Database 1 DOI | Accessibility |  |
|  | Query methodology for Database 1 | Transparency |  |
|  | In which of the following does the researcher share a query script for Database 1: | Accessibility |  |
|  | In what format are query documentation files shared for Database 1? | Accessibility |  |
|  | Does the shared query script for database 1 contain comments and/or notations for ease of reproducibility? | Transparency | 100% |
|  | Are query resources used throughout data collection proprietary or non-proprietary for database 1? | Accessibility |  |
|  | Does the author share the dataset metadata for database 1? | Transparency & Accessibility | 100% |
|  | Level of restriction for access to database 1 | Accessibility | 50% |
|  | Must one pay for access to database 1? | Accessibility | 100% |
|  | *Database Name 2* | *Accessibility* |  |
|  | *Database 2 DOI* | *Accessibility* |  |
|  | *Query methodology for Database 2* | *Transparency* |  |
|  | *In which of the following does the researcher share a query script for Database 2:* | *Accessibility* |  |
|  | *In what format are query documentation files shared for Database 2?* | *Accessibility* |  |
|  | *Does the shared query script for database 2 contain comments and/or notations for ease of reproducibility?* | *Transparency* |  |
|  | *Are query resources used throughout data collection proprietary or non-proprietary for database 2?* | *Accessibility* |  |
|  | *Does the author share the dataset metadata for database 2?* | *Transparency & Accessibility* | *100%* |
|  | *Level of restriction for access to database 2* | *Accessibility* | *33.33%* |
|  | *Must one pay for access to database 2?* | *Accessibility* | *100%* |
|  | *Database Name 3* | *Accessibility* |  |
|  | *Database 3 DOI* | *Accessibility* |  |
|  | *Query methodology for Database 3* | *Transparency* |  |
|  | *In which of the following does the researcher share a query script for Database 3:* | *Accessibility* |  |
|  | *In what format are query documentation files shared for Database 3?* | *Accessibility* |  |
|  | *Does the shared query script for database 3 contain comments and/or notations for ease of reproducibility?* | *Transparency* |  |
|  | *Are query resources used throughout data collection proprietary or non-proprietary for database 3?* | *Accessibility* |  |
|  | *Does the author share the dataset metadata for database 3?* | *Transparency & Accessibility* | *100%* |
|  | *Level of restriction for access to database 3* | *Accessibility* | *100%* |
|  | *Must one pay for access to database 3?* | *Accessibility* | *100%* |
|  | Does the researcher state a reason for not sharing a query script? | Accessibility | 96.67% |
|  | Please briefly explain the author's reasoning for not sharing a query script. | Transparency |  |
|  | For how many databases does the researcher fail to share a query script? | Transparency |  |
|  | Does the publication or shared query material(s) state cohort inclusion and exclusion criteria? | Transparency | 82.35% |
|  | States cohort inclusion and exclusion criteria in the following location: | Accessibility |  |
|  | Are inclusion and exclusion criteria operationalized using standard ontologies and vocabularies? | Transparency | 92.59% |
|  | States standard ontologies and vocabularies in the following location: | Accessibility |  |
|  | Procedure vocabulary adopted: | Transparency |  |
|  | Other procedure vocabulary adopted | Transparency |  |
|  | Diagnosis vocabulary adopted: | Transparency |  |
|  | Other diagnosis vocabulary adopted: | Transparency |  |
|  | Medication vocabulary adopted | Transparency |  |
|  | Other medication vocabulary adopted: | Transparency |  |
|  | Laboratory vocabulary adopted: | Transparency |  |
|  | Other laboratory vocabulary | Transparency |  |
|  |  |  |  |
| **Methods: Data Mining and Cleaning (19 items)** | | |  |
|  | Does the research involve natural language processing or text mining? | Transparency | 0.870 |
|  | Does the manuscript state the source of the text from which data was mined? | Transparency & Accessibility | 100% |
|  | In which of the following is the source text shared? | Accessibility |  |
|  | Does the publication clearly state processes for validating data mined via NLP and/or queried from a database? | Transparency |  |
|  | Please list all software applications used for text mining: | Transparency |  |
|  | Is the text mining software application proprietary or open? | Accessibility |  |
|  | Are the text mining materials shared in a format interoperable with systems other than the author's chosen software application? | Accessibility | 100% |
|  | In what format are text mining files shared? | Accessibility |  |
|  | Does the author state how data were cleaned? | Transparency | 90.91% |
|  | In which of the following does the author describe any data cleaning procedures? | Accessibility |  |
|  | Does the author state how data were merged? | Transparency | 93.94% |
|  | In which of the following does the author describe any data merging procedures? | Accessibility |  |
|  | Does the author or analyst use a patient primary identifier - such as SSN, UPI, or MRN - throughout merging besides FN, LN, DOB? | Transparency | 94.12% |
|  | Does the author state any clear process documented for cleaning messy or ambiguous records? | Transparency |  |
|  | In which of the following does the author describe any procedures for accounting for messy or ambiguous data? | Accessibility |  |
|  | Does the author state any clear process documented for accounting for missing data? | Transparency | 0.520 |
|  | In which of the following does the author describe any procedures for accounting for missing data? | Accessibility |  |
| **Methods: Data Analysis (15 items**) | | |  |
|  | Does the author share all code sufficient for data analysis? | Accessibility | 100% |
|  | Are all results listed within the manuscript's results section verifiable via the shared analysis scripts? | Transparency |  |
|  | Are all results listed within the manuscript's results section linked to commented and index sections of code? | Transparency |  |
|  | Does the shared analysis code contain comments and/or notation for ease of reproducibility? | Transparency |  |
|  | Does the author state analysis methodology and process? | Transparency | 67.65% |
|  | In which of the following does the author describe any data analysis procedures? | Accessibility |  |
|  | Does the author indicate the software used to develop the analysis code? | Transparency & Accessibility | 0.880 |
|  | Software used for data analysis | Transparency & Accessibility |  |
|  | Analysis software version number | Transparency & Accessibility |  |
|  | Analysis software operating system | Transparency & Accessibility |  |
|  | Is the analysis software proprietary or open? | Accessibility |  |
|  | Analysis file formats shared | Accessibility |  |
|  | Where is the finalized data analysis code shared? | Accessibility |  |
|  | What is the level of restriction for requesting the data analysis script? | Accessibility |  |
| **Data Sharing and Data Documentation (36 items)** | | |  |
|  | Is the finalized dataset shared? | Accessibility | 100% |
|  | Where is the finalized dataset shared? | Accessibility |  |
|  | If Other, where? | Accessibility |  |
|  | Is there a clear process for requesting the data? | Accessibility | 85.29% |
|  | Which of the following specifies a data request process? | Accessibility |  |
|  | If Other, where? | Accessibility |  |
|  | Does the journal of publication have a data sharing policy? | Accessibility | 56.25% |
|  | If yes, please input the data sharing url: | Accessibility |  |
|  | What is the level of restriction for requesting the data? | Accessibility | 29.63% |
|  | If other, please indicate level of restriction for requesting the data | Accessibility |  |
|  | Is there a charge associated with requesting the data? | Accessibility | 56% |
|  | Does the author share a readme file with each dataset? | Accessibility | 100% |
|  | Does the readme file contain clear documentation of dataset contents and related metadata? | Transparency |  |
|  | Does the author indicate an existing metadata standard they are applying within their metadata? | Transparency |  |
|  | Does the author document any form of adopted file naming convention? | Transparency |  |
|  | Are contents within the readme file organized through some form of index? | Transparency |  |
|  | Does the readme file contain an inventory of research data contents? | Transparency & Accessibility |  |
|  | Does the readme file document any associated licenses or rights of reuse for the associated research data and materials? | Accessibility |  |
|  | Does the readme file contain a project abstract or project description? | Transparency |  |
|  | Does the readme file state creation dates for all referenced files and datasets? | Transparency |  |
|  | Does the readme file reference related publications or supplementary materials? | Transparency |  |
|  | Does the readme file explain the relevant operating systems used throughout the creation of inventoried files? | Transparency |  |
|  | Does the readme file document the relevant software version numbers adopted throughout the research process? | Transparency |  |
|  | Does the readme file document limitations associated with the associated research methods or shared data? | Transparency |  |
|  | In which of the following locations are project or data limitations documented? | Accessibility |  |
|  | Does the author share a data dictionary for all datasets? | Transparency & Accessibility | 100% |
|  | In which of the following locations is the data dictionary shared? | Accessibility |  |
|  | Are all variables clearly intelligible to those within the clinical domain? | Transparency |  |
|  | Is the use of abbreviations within the data dictionary and associated data documentation limited? | Transparency |  |
|  | Are all abbreviations spelled out and defined in metadata? | Transparency |  |
|  | Are truncated variable titles spelled out in full within the data dictionary? | Transparency |  |
|  | Are all coded variable labels assigned clear definitions? | Transparency |  |
|  | Are all datasets and data documentation files available through the same centralized request mechanism(s)? | Accessibility |  |
